# Anterior chamber associated immune deviation used as a neuroprotective strategy in rats with spinal cord injury

**DOI:** 10.1371/journal.pone.0188506

**Published:** 2017-11-30

**Authors:** Beatriz Pineda-Rodriguez, Diana Toscano-Tejeida, Elisa García–Vences, Roxana Rodriguez-Barrera, Adrian Flores-Romero, Daniela Castellanos-Canales, Gabriel Gutierrez–Ospina, Laura Castillo-Carvajal, Esperanza Meléndez-Herrera, Antonio Ibarra

**Affiliations:** 1 Centro de Investigación en Ciencias de la Salud (CICSA), Facultad de Ciencias de la Salud; Universidad Anáhuac México Campus Norte. Avenida Universidad Anáhuac No. 46, Colonia Lomas Anáhuac, Huixquilucan Estado de México, México; 2 Departamento de Biología Celular y Fisiología, Instituto de Investigaciones Biomédicas, Universidad Nacional Autónoma de México, Ciudad de México, México; 3 Laboratorio de Ecofisiología Animal, Departamento de Zoología, Instituto de Investigaciones sobre los Recursos Naturales, Universidad Michoacana de San Nicolas de Hidalgo, Morelia, Michoacán, México; 4 Proyecto CAMINA A.C., Ciudad de México, México; University of South Florida, UNITED STATES

## Abstract

The inflammatory response is probably one of the main destructive events occurring after spinal cord injury (SCI). Its progression depends mostly on the autoimmune response developed against neural constituents. Therefore, modulation or inhibition of this self-reactive reaction could help to reduce tissue destruction. Anterior chamber associated immune deviation (ACAID) is a phenomenon that induces immune-tolerance to antigens injected into the eye´s anterior chamber, provoking the reduction of such immune response. In the light of this notion, induction of ACAID to neural constituents could be used as a potential prophylactic therapy to promote neuroprotection. In order to evaluate this approach, three experiments were performed. In the first one, the capability to induce ACAID of the spinal cord extract (SCE) and the myelin basic protein (MBP) was evaluated. Using the delayed type hypersensibility assay (DTH) we demonstrated that both, SCE and MBP were capable of inducing ACAID. In the second experiment we evaluated the effect of SCE-induced ACAID on neurological and morphological recovery after SCI. In the results, there was a significant improvement of motor recovery, nociceptive hypersensitivity and motoneuron survival in rats with SCE-induced ACAID. Moreover, ACAID also up-regulated the expression of genes encoding for anti-inflammatory cytokines and FoxP3 but down-regulated those for pro-inflamatory cytokines. Finally, in the third experiment, the effect of a more simple and practical strategy was evaluated: MBP-induced ACAID, we also found significant neurological and morphological outcomes. In the present study we demonstrate that the induction of ACAID against neural antigens in rats, promotes neuroprotection after SCI.

## Introduction

Spinal Cord Injury (SCI) is a degenerative disease that issues multiple events that have a great impact on life quality The global incidence rate is estimated in 23 cases per million inhabitants (179,312 cases per annum) [1, 2].

After the initial mechanical injury, several destructive events occur promoting a significant increase in the lesion size [3]. This condition promotes the release of large amounts of neural constituents and, thereby, the contact of these elements with the immunological system, inducing the activation of immune cells against neural antigens. This self-reactive response exacerbates the inflammatory reaction promoting a significant increase in the tissue destruction [4]. Inflammation in conjunction with the observed self-reactive response, will also damage healthy tissue [5, 6]. For this reason, numerous investigations have been carried out in order to propose strategies aimed at finding ways to modulate or to inhibit such immunological responses [6–10].

In the light of this investigation, arises the possibility of exploring new innovative strategies. Anterior chamber associated immune deviation (ACAID) refers to a phenomenon in which, immune-tolerance to antigens is achieved by the injection of the antigen into the eye’s anterior chamber (AC) [11]. Given the fact that an uncontrolled immune response is one of the fundamental mechanisms contributing to neural degeneration after SCI [12], it is possible that the induction of immune-tolerance to neural antigens using ACAID as a potential prophylactic therapy, could achieve a reduction or even a total inhibition of the immune response after SCI [13].

Extremely delicate target organs, like brain and eyes, could be damaged by an extensive inflammatory response. That is why such organs are endowed with particular mechanisms to protect themselves from this process [14]. Immunologic ignorance (peripheral tolerance to the antigens created in these organs) as well as the creation of immunosuppressive microenvironments, are part of the mechanisms by which ACAID could exert its immunomodulatory effect [14].

An important feature of ACAID is that regulatory cells are antigen specific, thereby, immune-tolerance to an antigen of interest, can be induced after inoculating it into the eye’s AC [11].

With this basis, we dare to construct the following hypothesis: if we inoculate neural constituents within the eye’s AC, we could induce immune-tolerance against neural proteins, this will decrease the inflammatory response and consequently reduce the tissue degeneration and therefore, improve functional recovery. The last mentioned, could be of great help for individuals with SCI because the better the neural recovery is, the better the quality of life will be.

At the moment, there is not a reliable strategy to provide the patients an efficient and prophylactic therapy. Induction of ACAID could be an interesting prophylactic approaching to be evaluated at clinical settings.

ACAID could also be proposed as a therapeutic strategy for other neurodegenerative diseases as Multiple sclerosis, Alzheimer´s disease and Amiotrophic lateral sclerosis. These diseases share a common pathological pattern; first, the establishment of an autoimmune-like response that leads to inflammation and second, the difficulty to resolve such inflammation. In these cases, a potential strategy to modulate the immune system could be of real benefit for patients. ACAID is capable of inducing antigen-specific immune tolerance and thus, it may prevent or reduce the establishment of autoimmune disease and /or promote the resolution of inflammation [13]. In this article we evaluate the effect of ACAID on neuroprotection and functional recovery in rats with SCI.

## Materials and methods

### Ethics statement

All animals were handled according to NIH guidelines for the management of laboratory animals. All the procedures were performed in accordance to the National Institutes of Health *Guide for the care and use of laboratory animals* and the Mexican Official Norm on the Principles of Laboratory Animal Care (NOM 062-ZOO-1999). The study was approved by the Institutional Animal Care and Use Committee and Institutional Review Board of Universidad Anahuac Mexico Norte (ID: 201210). All experiments were designed and reported according to the ARRIVE guidelines. In order to perform any procedure (SCI, AC-inoculation, immunization, DTH or euthanasia), animals were previously anesthetized by intramuscular injection with a mixture of ketamine (50 mg/kg) and xylazine (10 mg/kg).

### Animal care

Animals were matched for age and weight in each experiment and housed in pairs in a light and temperature-controlled room. To minimize stress, animals were handled daily at least once a day 7 days prior to the surgical procedure.

Sterile bedding and filtered water was replaced daily. Bladder emptying was performed by manual expression three times a day until automatic voidance was regained. During the first day after injury, the animals received a course of enrofloxacine (Marvel, Mexico City, Mexico) in their drinking water at an approximate dose of 64 mg/kg/d. All rats were carefully monitored for evidence of postsurgical complications. Animals with signs of infections were excluded from the study.

### Study design

In order to evaluate the effect of ACAID on neuroprotection and neurological recovery, three experiments were performed. In the first, we evaluated the induction of ACAID against a spinal cord extract (SCE, n = 6), myelin basic protein (MBP, n = 6) or ovalbumin (OVA, n = 6). In the second experiment, we evaluated the neuroprotective effect of SCE- induced ACAID. For this purpose, thirty day-old rats were inoculated into the anterior chamber either with SCE (n = 10) or PBS (n = 10). After 2 months, all the animals were subjected to a spinal cord contusion and then, evaluated for motor and sensitive recovery for 2 months. At the end of this period, animals were euthanized and the spinal cord was prepared to quantify the number of surviving motoneurons (n = 5) and the gene expression of some cytokines at the site of injury. Expression of genes encoding for some cytokines was also assessed in the spleen (n = 5). In the third experiment, we evaluated the neuroprotective effect of MBP- induced ACAID. In this case, we inoculated MBP (n = 8), OVA (n = 8) or PBS (n = 8) into the anterior chamber of thirty-day old rats. After 2 months the rats were subjected to SCI and then evaluated for a period of 2 months for motor and sensitive recovery. To the end of the study the animals were euthanized and the number of surviving motoneurons was quantified. All rats were set into an inverted cycle of light darkness 12/12, food and water was given ad libitum.

### Obtention of spinal cord extract

In order to obtain the SCE, 2 adult Sprague Dawley rats of 200–250 g weight were sacrificed and then, complete spinal cord (from the medulla bulb up to the cauda equina) was obtained. Two grams of macerated tissue, previously infused with protease inhibitor, were placed in an eppendorf tube along with 0.5 ml of extraction buffer and then centrifuged (25 000 rpm) for 45 minutes at 4 C°. Once centrifugation was over, supernatant was removed. To obtain highly associated membrane proteins, 1 ml of extraction buffer + triton 100 X was homogenized with a pistil and placed for 10 seconds in the vortex. After this, it was placed in the centrifuge again for 45 minutes (25000 rpm) at 4C. Afterwards, supernadant was removed and protein concentration was measured with the Bradford protein assay.

### Induction of ACAID

#### Theoretical bases of ACAID

ACAID starts with the injection of a specific antigen into the eye’s AC. There, the antigen-presenting cells (APC) F4/80+ capture the antigen and enter the bloodstream through the venous circulation, taking the antigen through the spleen and thymus. In the thymus, APC F4/80+ induce the generation of thymocites CD4-CD8-NK1.1+, which travel into the spleen. In the spleen, these cells, together with CD4-CD8-NK1.1+ thymocytes, form multicellular clusters with T NK cells, lymphocytes BCD1d+ and lymphocytes T CD8+ rich in TGF-beta and IL-10. The APCs, in the presence of TGF-beta and IL-10, are able to introduce the antigens into the cell and later, release them processed into the marginal area of the spleen where they interact with B cells. Activated B cells, then process and present those antigen fragments to the T CD8+ and CD4+ and turn them into regulatory T cells. This process leads to the activation of T cells which will be capable of suppressing a late inflammatory response [15].

#### Induction of ACAID

For the induction of ACAID, thirty-day old rats were anesthetized with an intra-peritoneal injection of ketamine/xylazine, and then one drop of lidocaine (1% dilution PISA laboratory, Mexico) was applied topically on each eye before injection to reduce corneal reflex [16]. Under a dissecting microscope, a 30-G disposable needle (BD Co., Mexico) attached to a manually controlled Hamilton syringe (701LTSYR, Hamilton Co., USA), was inserted into the AC and the aqueous humor was allowed to drain [17]. Approximately 10 microliters of PBS alone or containing antigens (50 micrograms of OVA or MBP), were injected into the AC of both eyes respectively for each group [18]. Rats receiving only PBS via intracameral injection were used as controls. A sterile patch with saline was applied in both eyes as an environmental protection until the animals recovered after injection.

### Evaluation of ACAID induction

ACAID development was evaluated by using the delayed-type hypersensitivity (DTH) assay. For this purpose, in the first step we immunized the animals of each group with the corresponding antigen (SCE, MBP or OVA). Afterwards, the DTH assay was performed.

#### Immunization

On day 7 post AC injection, rats were immunized subcutaneously at the interscapular space with 150 micrograms of SCE, MBP or OVA in PBS. Antigens were emulsified 1:1 in complete Freund’s adjuvant (CFA; Sigma) containing 0.5 mg/ml Mycobacterium tuberculosis. Each animal received a total volume of 200 microliters. Immunization was performed under anesthetizing conditions.

#### DTH assay

On day 14 post AC injection (which is 7 days after subcutaneous immunization), under anesthetizing conditions, antigens (SCE, MBP or OVA) were injected (400 micrograms/20 microliters) intradermally into the left ear pinna (experimental ear), and PBS alone was injected into the right ear pinna (as an internal control) [16, 18]. Both ears thickness were measured before and 48hr after intradermal injection, using a Mitutoyo digital engineer’s micrometer (Mitutoyo Co., Mexico). The difference in ear swelling was analysed as an indicator of a DTH response using the following equation: specific ear swelling = [(48hr measurement– 0hr measurement) for left ear–(48hr measurement– 0hr measurement) for right ear] [19].

### Spinal cord injury

Adult female Sprague-Dawley rats weighing between 230–250g were subjected to moderate SC contusion. The animals were anesthetized by intramuscular injection with a mixture of ketamine (50 mg/kg, Probiomed, Mexico City, Mexico) and xylazine (10 mg/kg, Fort Dodge Laboratories, Fort Dodge, Iowa). The skin was opened in layers and a laminectomy was performed at the T9 level. The injury was performed by direct contusion of the exposed spinal cord using the IH-0400 impactor at 200 Kdyn, in order to achieve a moderate SCI.

The functional recovery of all groups was assessed by the BBB locomotor scale [20]

### Evaluation of neurological recovery

For the evaluation of motor and sensitive recovery along the first eight weeks, two tests were used; the BBB test which was performed weekly and the von Frey Hair that was performed monthly. Results were evaluated by blind observers.

#### Assessment of motor recovery

Behavioral recovery was assessed every week after spinal cord contusion using the Basso, Beattie & Bresnahan (BBB) open-field test of locomotor ability [20]. Three separate blinded observers evaluated all animals and the average of the three scores was used.

#### von Frey Hair test

The rats were placed in a clear acrylic glass enclosure on an elevated metal mesh floor and allowed to acclimate to the new environment for 15 min. The paw-withdrawal response to non-noxious mechanical stimuli was recorded using an Electronic von Frey Anesthesiometer (IITC Life Science, Inc., Woodland Hills, CA) [21]. The plantar surface of each hindpaw of the rats was stimulated with von Frey plastic filaments perpendicularly, and the maximum pressure required to elicit a response was automatically registered. Three scores for each paw were recorded and averaged. This motor analysis was performed before spinal cord injury to ensure that the animals showed normal responses and it was repeated after surgery for each group one month and two months later.

### Semiquantitative gene expression

The gene expression of Interleukin 4 (IL-4), Interleukin 10 (IL-10), Transfo rming growth factor-beta (TGFβ), Tumor necrosis factor alpha (TNFα), Interferon gamma (INFγ), Forkhead box P3 (FoxP3), and Hypoxanthine phosphoribosyl transferase (HPRT, housekeeping gene) was determined by qRT-PCR, 60 days after injury. Total RNA (RNAt) was isolated from a 1.0 cm-long sample taken from the injury site of the spinal cord (0.5cm caudal/0.5cm rostral) or 3g of spleen using the Trizol method (Invitrogen, Carlsbad, CA, USA). Then, cDNA was synthesized from 2 μg of total RNA using the Superscript II transcriptase enzyme and Oligo dT (Invitrogen, Carlsbad, CA, USA). The primers were designed by Custom Primers OligoPerfect^TM^ designer (http://tools.invitrogen.com) and confirmed by Primer analysis software (Oligo) (Molecular Biology Insights, Inc). The forward (F) and reverse (R) primers, amplicon size, and GeneBank entry numbers are listed in Table 1.

**Table 1:** PCR primers.

Gene	Reference sequence number	Sequence	Product length
**Hypoxanthine guanine phosphoribosyltransferase. HPRT**	NM012583	Forward 5´AAGCTTGCTGGTGAAAAGG3´ Reverse 3´CAAAGCCTAAAAGACAGCGG5´	192 pb
**Interferon gamma. IFNɣ**	NM138880	Forward 5’AACCAGGCCATCAGCAACA3’ Reverse 3’TCTGTGGGTTGTTCACCTCG5’	128 pb
**Tumor necrosis factor alpha. TNF α**	NM012675	Forward 5’CTCTTCTGTCTACTGAACT3’ Reverse 3’GAGAAGATGATCTGAGTGTG5’	115 pb
**Interleukin 4. IL4**	NM201270	Forward 5’GGCTTCCAGGGTGCTTCGC3’ Reverse 3’GTGGACTCATTCACGGTGCA5’	150 pb
**Interleukin 10. IL10**	X60675	Forward 5’GGGGTGACAATAACTGCAT3’ Reverse 3’GGGGCATCACTTCTACCAT5’	216 pb
**Transforming growth factor, beta 3. TGFb3**	NM013174	Forward 5´CCCAACCCCAGCTCCAAGC3´ Reverse 3´AGCCACTCTGCGGTGCCTCG5´	132 pb
**Forkhead box P3. Foxp3**	NM110825	Forward 5´CTGCCTGTGCTTAGGAGAC3´ Reverse 3´CATCACTGGCTTTCGCGTA5´	156 pb

The reactions were performed with the FastStart Essential DNA Green Master kit (Roche, Diagnostics, Indianapolis, USA). The amplification was detected with a LigthCycler 96 instrument (Roche Diagnostics, Indianapolis, USA).

Relative concentrations were calculated by the Cq method (i.e., the cycle number in which the exponential amplification of the template begins) running the second derivative. The average value of each sample was obtained. The expression value from each analyzed gene was compared to that of the housekeeping gene by assigning a value equal to one, to the latter for the normalization of the expression.

### Number of surviving ventral horn neurons

Five rats from each group were anaesthetized and perfused intra-aortically with 100 ml of PBS, pH 7.4 plus heparin (1%) at 4°C, followed by 400 mL of a fixative solution (4% paraformaldehyde in PBS, pH 7.4 at 4°C). One centimeter of tissue at the lesion site of spinal cord (0.5 cm caudal/rostral) was removed and incubated for 2 h in the same fixative solution and then cryoprotected in a 30% sucrose solution for at least 3 days. Afterwards, 3 sequential cryosections 10 μm thick were cut at 0.5, 1, 2 and 3 mm caudal to rostral from the epicenter of injury. Haematoxylin & Eosin-stained sections were analyzed for surviving ventral horn neurons. The number of surviving neurons were confirmed and counted by the presence of Nissl substance, euchromatic nucleus and nucleolus [22]. The number of neurons in each rat was given by the average number of cells counted in its three sequential sections.

### Statistical analysis

Data was analyzed using the GraphPad Prism 3.0 software and presented as mean ± standard deviation (SD). DTH response was evaluated using a One way ANOVA followed by Tukey test. Motor recovery was evaluated using a two-way ANOVA for repeated measures. von Frey test and the number of surviving motoneurons were analyzed using a One way ANOVA followed by Tukey test. Cytokine gene expression was analyzed using a Student T test. Differences of p≤ 0.05 were considered statistically significant.

## Results

### Delivery of SCE or MBP into the AC developed ACAID

**Fig 1:**
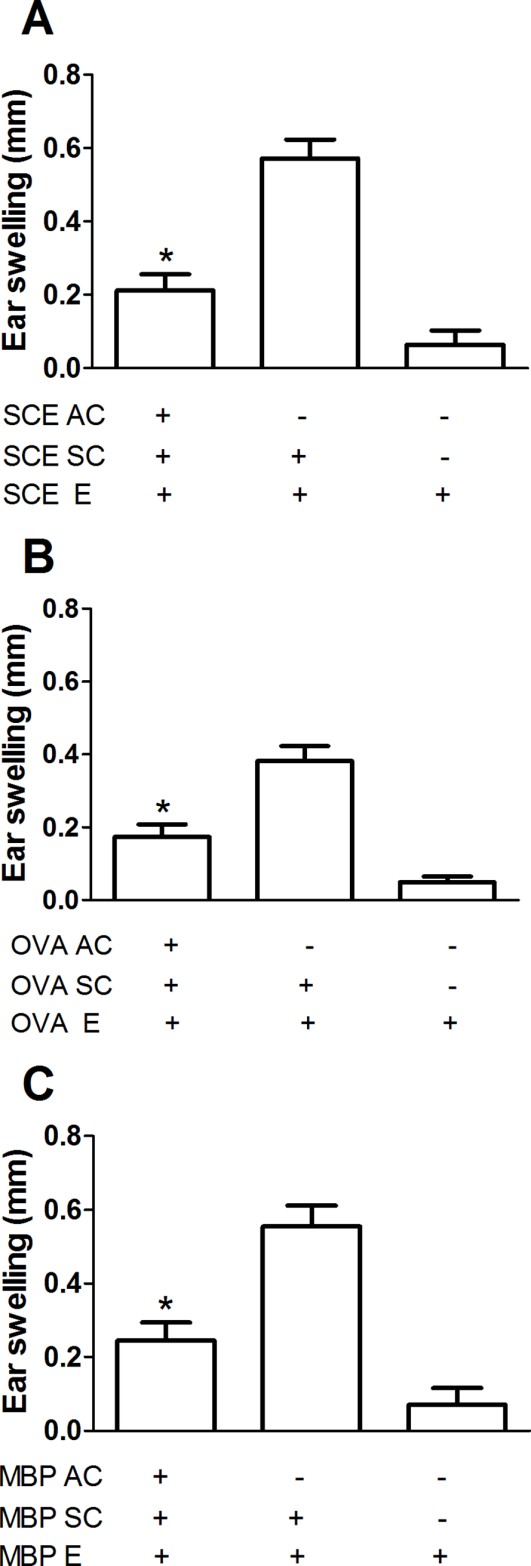
Spinal cord extract, myelin basic protein and ovalbumin (OVA) were capable of inducing ACAID. DTH response was inhibited in ACAID-induced groups relative to control rats that did not receive the AC injection. Bars represent the mean ± SD of 6 rats. This is one representative of 3 experiments. * Different from the groups with no inoculation into the AC, p < 0.05; One way ANOVA followed by Tukey test. SCE, spinal cord extract, OVA, ovalbumin; MBP, myelin basic protein; AC, inoculation into the anterior chamber; SC, subcutaneous immunization; E, ear priming.

In the first step, we attempted to induce tolerance against a spinal cord extract (SCE) or the most immunogenic neural constituent, myelin basic protein (MBP). As a control antigen was required for the second experiment of this work, we also induced tolerance against ovalbumin (OVA), a CNS-irrelevant protein. The main objective of this step was to demonstrate the development of ACAID against these antigens. As ACAID is characterized by the inhibition of delayed–type hypersensitivity (DTH) reactions to the AC-injected antigens [23], we performed the DTH assay for each antigen to show the development of ACAID. Fig 1 shows that rats primed in the AC with SCE (Fig 1A), OVA (Fig 1B) or MBP (Fig 1C) did develop immune tolerance that was confirmed by significant suppression of SCE-, OVA- or MBP- specific DTH responses compared with control rats that did not receive the AC injection (p < 0.05, F = 163.3 and DF = 2 for SCE, 123 and 2 for MBP and 28.33 and 2 for OVA comparisons; One Way ANOVA followed by Tukey post-hoc test).

### Injection of SCE into the AC promoted better neurological and morphological recovery after spinal cord injury

**Fig 2:**
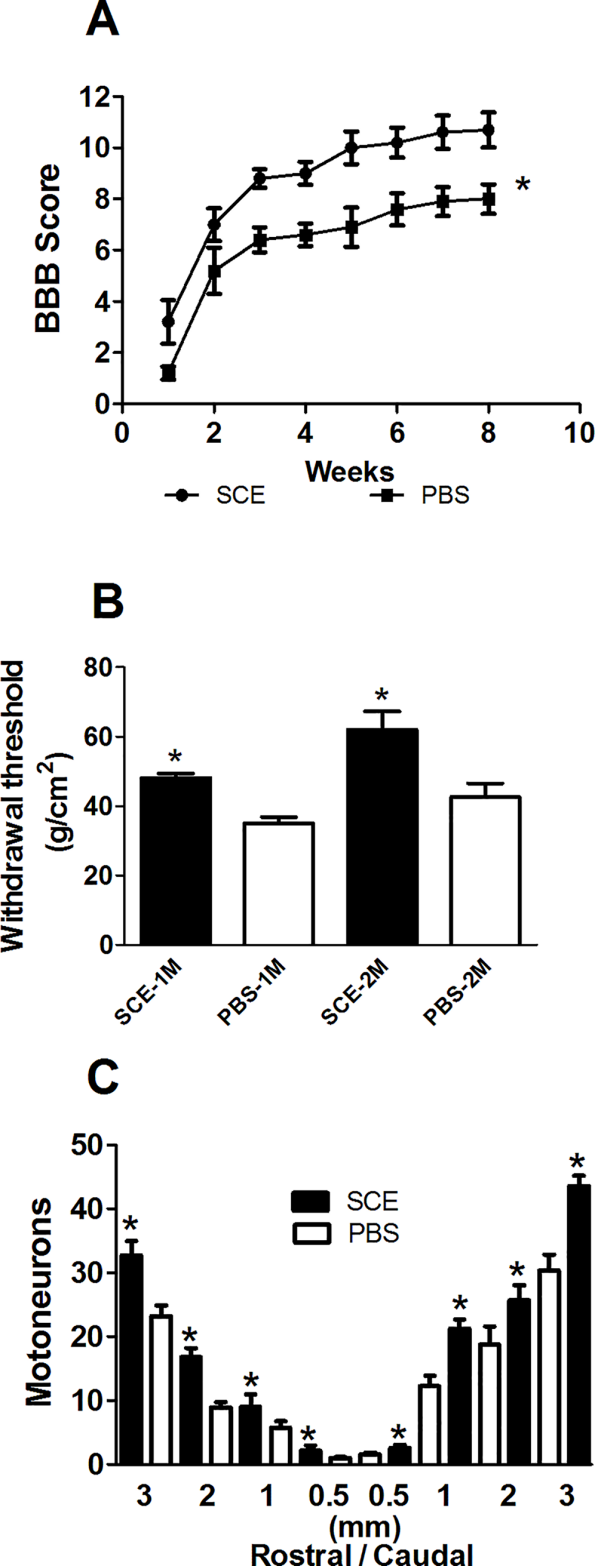
SCE-induced ACAID promoted functional and morphological improvement. Rats with SCE-induced ACAID presented a significant improvement in motor recovery (A), each point represents the mean ± SD of 10 rats. * Different from PBS group (p<0.05, two-way ANOVA for repeated measures). In the same way, ACAID improved the nociceptive hypersensitivity (B), bars represent the mean ± SD of 10 rats. * Different from PBS, p< 0.05; Student T test. Finally, SCE-induced ACAID promoted a better neuron survival (C). Bars represent the mean ± SD of 5 rats. * Different from PBS, p< 0.05; One Way ANOVA followed by Tukey test. SCE, spinal cord extract; PBS, phosphate buffer solution.

In order to test the effect of ACAID on neurological recovery, in the first experiment, we evaluated the motor performance of SCI-rats subjected to SCE-induced ACAID (n = 10) compared to the one observed in control animals (n = 10, SCI-rats receiving an injection of PBS into de AC). From the beginning to the end of the study, rats with SCE-induced ACAID showed the best motor recovery (Fig 2A; p<0.05, F = 48.72, DF = 7; Two-factor ANOVA for repeated measures). Seven days after injury they presented a significant improvement of locomotion (3.2 ± 0.8, mean ± SD) as compared to control ones (1.2 ± 0.2, p = 0.05, Mann-Whitney U test). In the same way, to the end of the follow-up, the rats with SCE-induced ACAID showed a significant increase of motor recovery (10.7 ± 0.6 vs 8 ± 0.5). As SCI and the consequent inflammatory reaction, trigger nociceptive hypersensitivity [24], we additionally evaluated, in the same animals, the development of mechanical hypersensitivity (MH). In this case, the lower is the damage, the less is the MH and thereby, the withdrawal threshold is greater (the animal needs higher levels of pressure to stimulate withdrawal response). Therefore, hind paw MH was assessed by measuring withdrawal threshold to mechanical stimulation with von Frey filaments. Compared to controls, the mechanical withdrawal threshold in the SCE-induced ACAID group was significantly increased at both 1 (48.18 ± 1.15 vs 34.97 ± 1.8; mean ± SD; p < 0.05, F = 2.6, DF = 5; student T test) and 2 (62.0 ± 5.2 vs 42.65 ± 3.9; p< 0.05, F = 1.8, DF = 5) months after SCI (Fig 2B). Finally, we investigated if the neurological recovery observed in SCE-induced ACAID group was associated to a preservation of neural tissue. For this purpose, the total number of surviving motoneurons at different levels of the SC was quantified. Fig 2C shows that at all levels, SCE-induced ACAID originated a higher number of surviving motoneurons as compared to control animals (p < 0.05, F = 67.03, DF = 15; One Way ANOVA followed by Tukey test).

### SCE-induced ACAID favorably changed the microenvironment observed in the site of injury and spleen

**Fig 3:**
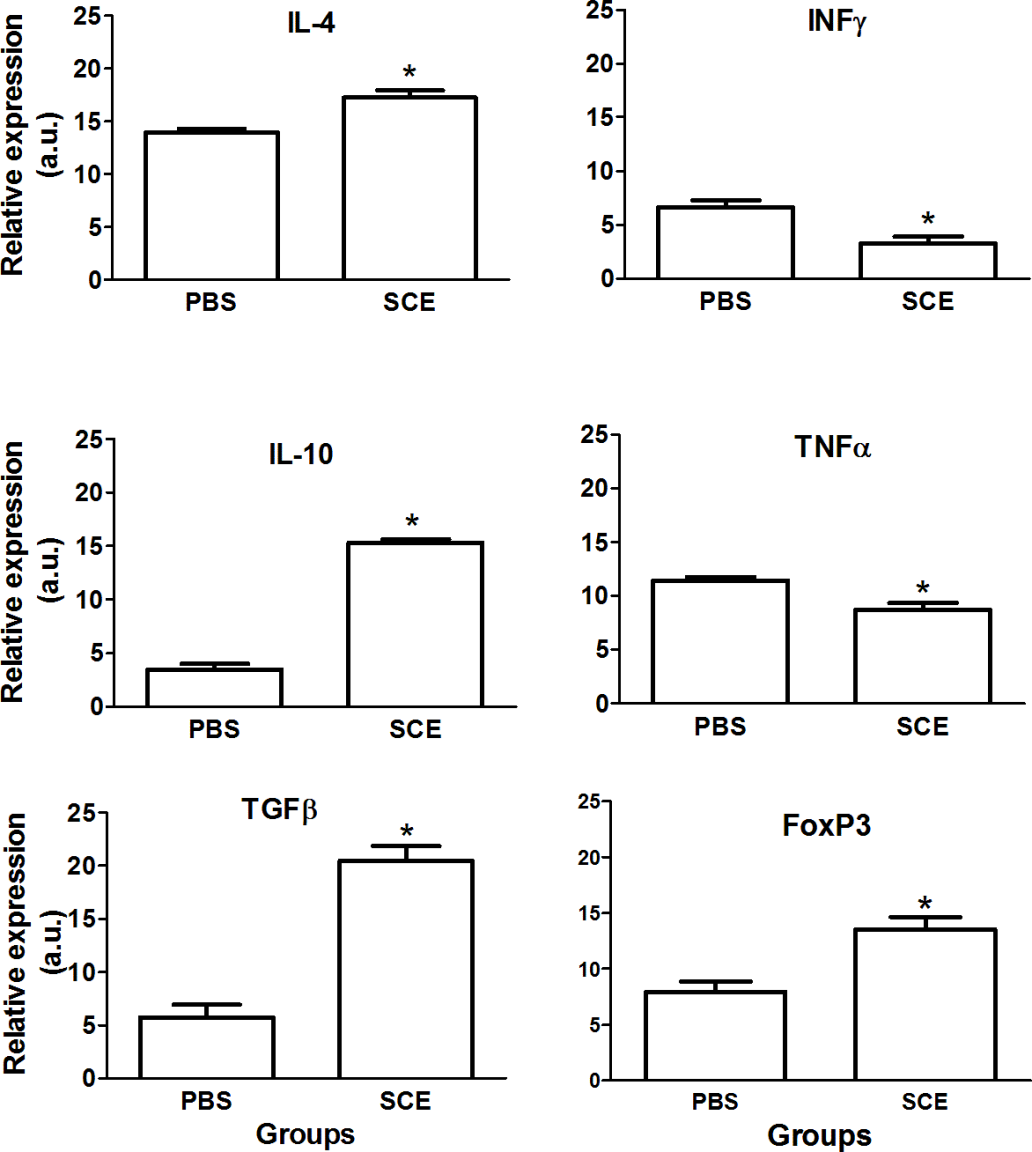
SCE-induced ACAID favorably changed the microenvironment at the site of injury. There was a significant increase in the expression of the genes encoding for anti-inflammatory cytokines and FoxP3 and a reduction in those encoding for pro-inflammatory cytokines. Bars represent the mean ± SD of 5 rats. This is one representative of 3 experiments. * Different from PBS, p< 0.05; Student T test. SCE, spinal cord extract; PBS, phosphate buffer solution.

In an attempt to elucidate the origin of the favorable effect observed in rats with SCE-induced ACAID, we now evaluated the expression of some genes encoding for pro-inflammatory (INFƔ and TNFα) or anti-inflammatory (IL-4, IL-10 and TGFβ) cytokines. Fig 3 shows that there is a significant increase of IL-4 (17.25 ± 0.7 vs 13.95 ± 0.3; mean ± SD, p < 0.05, F = 4.5, DF = 2; Student T test), IL-10 (15.29 ± 0.3 vs 3.5 ±0.5; p< 0.05, F = 2.1, DF = 2; Student T test) and TGFβ (20.53 ± 1.4 vs 5.71 ± 1.2; p <0.05, F = 1.3, DF = 2; Student T test) however, we found a reduction of INFƔ (3.29 ± 0.6 vs 6.64 ± 0.6; p< 0.05, F = 1.1, DF = 2; Student T test) and TNFα (8.72 ±0.6 vs 11.42 ± 0.3; p< 0.05, F = 3.3, DF = 2; Student T test) in the site of injury of rats with SCE-induced ACAID. On the other hand, taking into account that regulatory T lymphocytes are the cells that mainly mediate ACAID [25], we investigated the expression of the gene encoding for forkhead box P3 (FoxP3), a transcription factor involved in the development and function of regulatory T cells [26]. Fig 3 shows that FoxP3 is significantly increased in rats with SCE-induced ACAID (13.54 ± 1.1 vs 7.9 ± 0.9; p < 0.05, F = 1.3, DF = 2; student T test).

**Fig 4:**
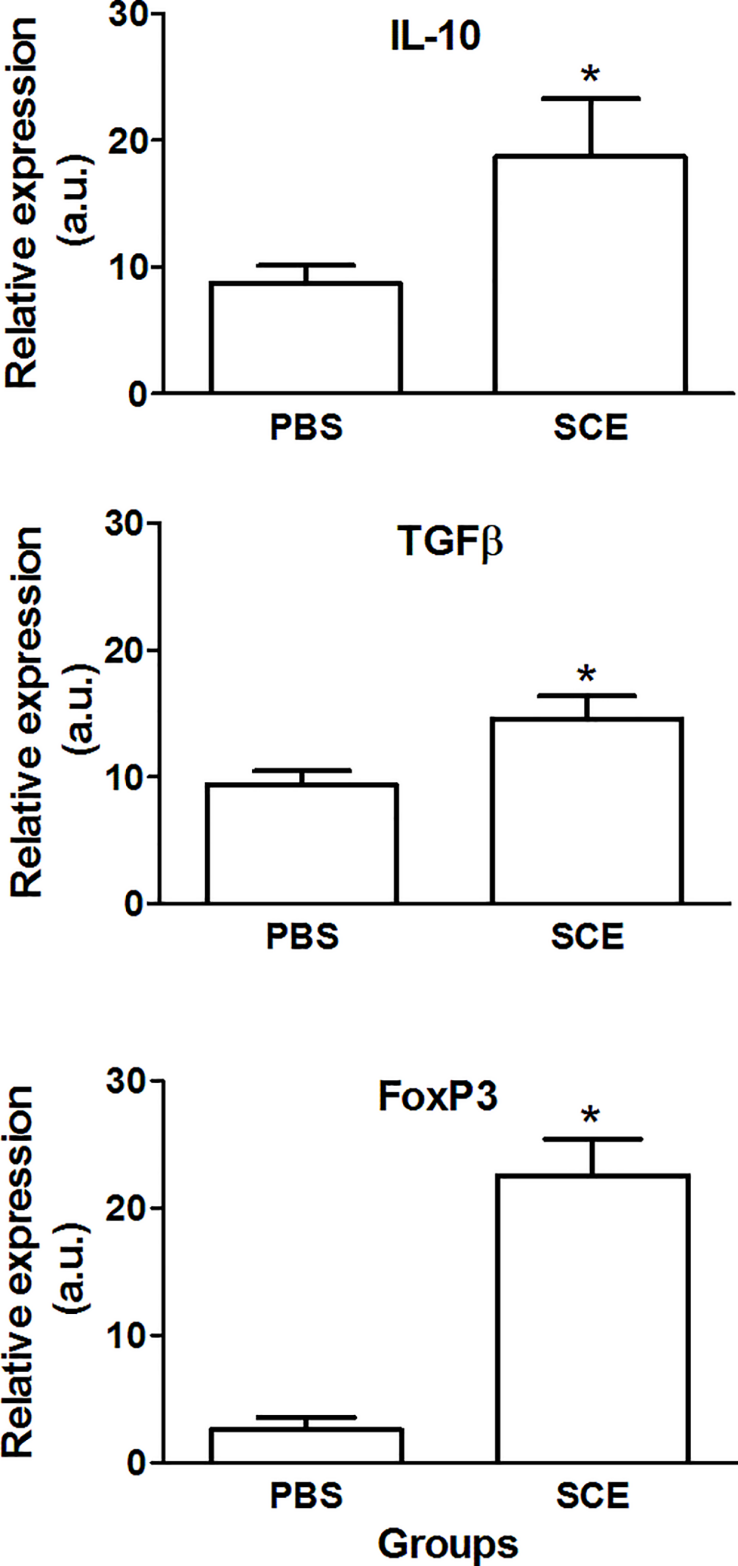
SCE-induced ACAID also changed the microenvironment observed in the spleen. ACAID provoked a significant increase of anti-inflammatory cytokines and FoxP3. Bars represent the mean ± SD of 5 rats. This is one representative of 3 experiments. * Different from PBS, p< 0.05; Student T test. SCE, spinal cord extract; PBS, phosphate buffer solution.

Finally, we also explored the microenvironment generated by SCE-induced ACAID in the spleen. In this case, we found also a significant increase in the expression of IL-10 (18.72 ± 4.6 vs 8.73 ± 1.4; p< 0.05, F = 11, DF = 2; Student T test), TGFβ (14.53 ± 1.9 vs 9.38 ± 1.1; p< 0.05, F = 3, DF = 2; Student T test) and FoxP3 (22.54 ± 2.9 vs 2.59 ± 1; p< 0.05, F = 8.2, DF = 2; Student T test) genes (Fig 4).

### Myelin basic protein-induced ACAID also improved neurological and morphological outcomes after SCI

**Fig 5:**
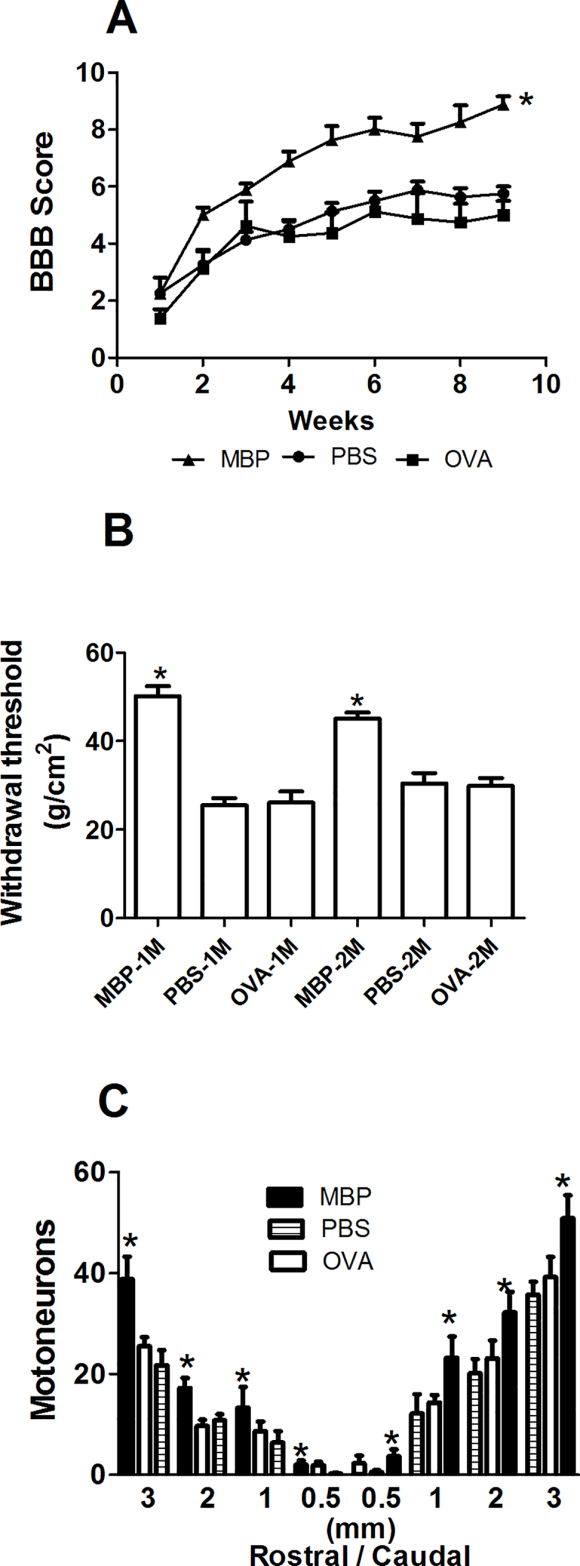
MBP-induced ACAID promoted functional and morphological improvement. The motor recovery of rats with MBP-induced ACAID was significantly better than the one observed in the control group (A), each point represents the mean ± SD of 8 rats. * Different from PBS and OVA groups (p<0.05, ANOVA for repeated measures). ACAID also improved the nociceptive hypersensitivity (B), bars represent the mean ± SD of 8 rats. * Different from PBS and OVA, p< 0.05; One way ANOVA followed by Tukey test. MBP-induced ACAID provoked a better neuron survival (C). Bars represent the mean ± SD of 4 rats. * Different from PBS and OVA groups, p< 0.05; One Way ANOVA followed by Tukey test. MBP, myelin basic protein; PBS, phosphate buffer solution, OVA, ovalbumin.

MBP is the most immunogenic protein in the CNS, thereby, it is the neural constituent capable of inducing the more prominent autoreactive response after SC injury [27]. MBP is an easily obtainable protein, and the induction of ACAID with this neural constituent, could be a practical and efficient therapeutic strategy after SCI. Therefore, we now investigated the effect of MBP-induced ACAID on neurological and morphological recovery of rats with SCI. In the same experiment, we also wanted to elucidate if the protective effect induced by ACAID is antigen-specific and, thereby, the induction of ACAID to a CNS-irrelevant antigen was not useful to promote neuroprotection. Thus, we induced ACAID to MBP (n = 8) or ovalbumin (n = 8) in 30 days-old rats. We also used animals inoculated only with PBS as controls (n = 8). Fig 5A shows that MBP-induced ACAID promoted a motor recovery that was significantly better (9.0 ± 0.3; mean ± SD, p <0.05, F = 103, DF = 8; ANOVA for repeated measures followed by Bonferroni´s test) than the one presented by OVA-induced ACAID (5.0 ± 0.5) or PBS-inoculated rats (5.8 ± 0.3). The evaluation of the hind paw MH, demonstrated that, the mechanical withdrawal threshold in the MBP-induced ACAID group was significantly increased at both, 1 (50.06 ± 2.3 vs 26.09 ± 2.5 and 25.47 ± 1.6; MBP, OVA and PBS respectively, mean ± SD) and 2 (45.06 ± 1.3 vs 29.84 ± 1.8 and 30.47 ± 2.3) months after SCI (Fig 5B; p < 0.05, F = 23.1, DF = 5; One way ANOVA followed by Tukey test) as compared to OVA-induced ACAID or PBS-inoculated groups. Finally, MBP-induced ACAID originated a higher number of surviving motoneurons as compared to OVA-induced ACAID or PBS-inoculated animals (Fig 5C; p < 0.05, F = 7.9, DF = 2; One Way ANOVA followed by Tukey test).

## Discussion

Spinal cord injury unleashes a cascade of auto-destructive phenomena that damage first, the adjacent injury tissues, and lately propagates beyond.

Immune response -when it is not regulated- might be one of these harmful events capable of promoting an important axonal damage and consequently a rapidly and increased loss of motor and sensitive function [6, 28]. This singular event, initiates as a nonspecific inflammatory response that rapidly becomes into a specific reaction against neural constituents, amplifying inflammation and thus, tissue damage [5, 29]. Several therapeutic strategies have been investigated in order to inhibit [29–31] or modulate [8, 32] this auto-reactive response. Nevertheless, only some studies have succeeded in obtaining promissory results [8, 33, 34]. That is why, the search for finding the best therapeutic strategy continues being the topic of research for numerous investigations. In the light of this goal, we envisioned the use of an innovative, practical and even preventive approach to promote neuroprotection after SCI. The development of ACAID to neural constituents could diminish and/or prevent, the inflammatory and auto-reactive phenomena observed after SCI. In this way, this strategy could reduce the damage to neural tissue, promoting then better neurological recovery.

At the moment, induction of ACAID as a therapeutic strategy has only been explored in animal models with experimental autoimmune encephalomyelitis (EAE). This is an animal model designed for studying multiple sclerosis, one of the CNS-autoimmune diseases that humans present [35]. EAE can be induced by immunizing susceptible animals with MBP. Myelin basic protein is capable of inducing antigen-specific immune tolerance after induction of ACAID [18]. Previous studies have shown that ACAID, induces a significant delay in the EAE onset, as well as a slower progression of the disease or even its total inhibition [25]. In the present work we demonstrated that SCE and MBP are capable of inducing ACAID and thereby, tolerance. This was supported by the results observed in the DTH-assays, where the inoculation of these antigens into the AC, suppressed the DTH response against them. Therefore, we thought that the development of ACAID to these antigens could promote beneficial effects after SCI.

The results presented here, support that the induction of ACAID to SCE is capable of promoting tissue protection and a neurological recovery superior to the one observed in the control group of animals.

These beneficial effects could be the result of the immune-modulation promoted by ACAID. In the site of injury, the animals with SCE-induced ACAID presented a microenvironment that was predominantly anti-inflammatory. Moreover, there was a significant reduction of pro-inflammatory cytokines, these data are in accordance with the results obtained in previous studies where ACAID induced the release of anti-inflammatory cytokines and suppressed those with inflammatory effect [25, 36–38]. In the present work, the microenvironment generated after ACAID, probably contributed to reduce some of the destructive events observed after SCI -including inflammation- and thus, the damage to neural tissue. The latter was corroborated when we evaluated the number of surviving motoneurons at the SC; the animals with SCE-induced ACAID presented a significant survival of this neurons that was significantly higher than the one observed in our control rats.

On the other hand, it has been shown that the ACAID-induced regulatory T cells (Tregs) suppress the progression of EAE, indicating an important role of these cells in the amelioration of the disease [25]. In our work, the microenvironment generated by SCE-induced ACAID at the site of injury, also presented a significant increase in the expression of the gene that encodes for FoxP3, the transcription factor involved in the development and function of Tregs. This finding supports that there was a significant increase in Tregs and thus, it is quite feasible to think that after SCI, Tregs prevented the activation of naïve T cells or inhibited the function of autoreactive T cells against neural antigens promoting then neuroprotection.

The immunomodulatory effects generated by ACAID were also corroborated in the spleen. According to previous studies, the presence of TGF-β and IL-10 in the spleen after induction of ACAID, is relevant to promote the development of Tregs [39]. In this regard, we found a significant increase in the expression of the genes encoding for these cytokines in the spleen of rats with SCE-induced ACAID. Furthermore, the expression of the FoxP3 gen, was also increased at this site. These observations provide evidence about the adequate development of ACAID but also explain, in some way, the increase of Tregs in both, spleen and the site of injury.

The results of this investigation showed that, the inoculation of SCE into the AC, is capable of improving motor function, however, it was also proficient to diminish the mechanical hypersensitivity observed after SCI. This is a relevant finding, since hypersensitivity could develop allodynia (non-noxious stimuli become noxious) or hyperalgesia (noxious stimuli become more noxious), different kinds of neuropathic pain that can disturb behavioral function and reduce the quality of life [40]. Since no cure is currently available for this type of chronic pain, the sensitive outcome observed in rats with SCE-induced ACAID should also be considered as a promissory benefit for individuals with SCI.

On the other hand, MBP-induced ACAID was also useful for improving neurological function. This is also an important finding, as the use of only MBP for inducing ACAID, could be the best strategy for this therapeutic approach. MBP is by now, an easily obtainable neural constituent that is commercially offered. Moreover, as MBP is a highly phylogenetically conserved protein [41, 42], it could be purified from animals and then used in humans to induce ACAID. Other investigations have already demonstrated the beneficial effects of ACAID when it is induced with only one protein. Induction of ACAID with oligodendrocyte glycoprotein (MOG) has promoted amelioration of EAE in mice [25].

The second experiment performed in rats with MBP-induced ACAID also demonstrated that the beneficial effects induced by ACAID are antigen specific since OVA-induced ACAID was not capable of promoting any positive effect after SCI. This was supported by the evaluations performed in animals with OVA-induced ACAID, where there was not any significant improvement in motoneuron survival or motor recovery.

Finally, we demonstrated that induction of tolerance to neural antigens could be a promising strategy to promote neuroprotection after SCI. Nevertheless, it is important to assess the cost-benefit of this approach as the inhibition of immune response could neglect the restoration of neural tissue. Whit this regard, autoreactive responses against neural antigens and inflammation by itself, have been proposed as relevant players in the way of promoting neural protection and restoration [32, 34, 43, 44]. The benefits provided by the immune system after SCI, have already been well documented. For instance, inflammatory cells clean cell debris and neurotoxic molecules. These events are orchestrated by microglia which also recruits systemic monocytes and neutrophils to the site of injury [45]. Lymphocytes also play a relevant role since T cells secrete trophic factors with neuroprotective and restorative effects [46]. In general terms, the presence of immune cells at the site of injury is also an indispensable element since, these cells are responsible for cleaning, protecting and even restoring the neural tissue [32, 33, 47]. Induction of ACAID could limit or even inhibit the beneficial effects provided by immune system. Therefore, the use of this strategy should continue to be carefully studied so that in the future, pre-clinical studies show sufficient evidence to implement this therapeutic action clinically in a devastating disease.
